# Neurophysiological Face Processing Deficits in Patients With Chronic Schizophrenia: An MEG Study

**DOI:** 10.3389/fpsyt.2020.554844

**Published:** 2020-09-03

**Authors:** Naotoshi Ohara, Yoji Hirano, Naoya Oribe, Shunsuke Tamura, Itta Nakamura, Shogo Hirano, Rikako Tsuchimoto, Takefumi Ueno, Osamu Togao, Akio Hiwatashi, Tomohiro Nakao, Toshiaki Onitsuka

**Affiliations:** ^1^Department of Neuropsychiatry, Graduate School of Medical Sciences, Kyushu University, Fukuoka, Japan; ^2^Medical Corporation Seiryokai, Mimamigaoka Hospital, Fukuoka, Japan; ^3^Department of Psychiatry, Harvard Medical School, Boston, MA, United States; ^4^Division of Clinical Research, National Hospital Organization, Hizen Psychiatric Medical Center, Saga, Japan; ^5^Center for Health Sciences and Counseling, Kyushu University, Fukuoka, Japan; ^6^Department of Molecular Imaging and Diagnosis, Graduate School of Medical Sciences, Kyushu University, Fukuoka, Japan; ^7^Department of Clinical Radiology, Graduate School of Medical Sciences, Kyushu University, Fukuoka, Japan

**Keywords:** schizophrenia, magnetoencephalography, M170, human faces, fusiform gyrus

## Abstract

**Background:**

Neuropsychological studies have revealed that patients with schizophrenia (SZ) have facial recognition difficulties and a reduced visual evoked N170 response to human faces. However, detailed neurophysiological evidence of this face processing deficit in SZ with a higher spatial resolution has yet to be acquired. In this study, we recorded visual evoked magnetoencephalography (MEG) and examined whether M170 (a magnetic counterpart of the N170) activity deficits are specific to faces in patients with chronic SZ.

**Methods:**

Participants were 26 patients with SZ and 26 healthy controls (HC). The M170 responses to faces and cars were recorded from whole-head MEG, and global field power over each temporal cortex was analyzed. The distributed M170 sources were also localized using a minimum-norm estimation (MNE) method. Correlational analyses between M170 responses and demographics/symptoms were performed.

**Results:**

As expected, the M170 was significantly smaller in the SZ compared with the HC group in response to faces, but not to cars (faces: p = 0.01; cars: p = 0.55). The MNE analysis demonstrated that while the M170 was localized over the fusiform face area (FFA) in the HC group, visual-related brain regions other than the FFA were strongly activated in the SZ group in both stimulus conditions. The severity of negative symptoms was negatively correlated with M170 power (rho = −0.47, p = 0.01) in SZ. Within HC, there was a significant correlation between age and the M170 responses to faces averaged for both hemispheres (rho = 0.60, p = 0.001), while such a relationship was not observed in patients with SZ (rho = 0.09, p = 0.67).

**Conclusion:**

The present study showed specific reductions in the M170 response to human faces in patients with SZ. Our findings could suggest that SZ is characterized by face processing deficits that are associated with the severity of negative symptoms. Thus, we suggest that social cognition impairments in SZ might, at least in part, be caused by this functional face processing deficit.

## Introduction

Social deficits caused by psychiatric disorders can be quite severe. Patients with schizophrenia (SZ) suffer from severe psychosocial dysfunctions and the substantial burden of the disease affects patients worldwide (1). The employment rate is low for patients with SZ (2), and social exclusion is commonly experienced (3). These dysfunctions could be attributed to impairments in social cognition, which is, at least in part, caused by face processing deficits (4). Therefore, it is important to investigate the neural basis of face recognition deficits in patients with SZ.

Neuropsychological studies have revealed that patients with SZ have facial recognition difficulties (e.g., 5–7). Neurophysiological approaches have also detected facial recognition deficits in SZ. The visually evoked N170 component is a negative potential recorded using electroencephalography (EEG) at occipitotemporal electrodes at around 170 ms after stimulus onset and reflects the early phase of face processing (8). A face-sensitive N170 reduction has been repeatedly reported in patients with SZ (9–11). Moreover, it has been suggested that this reduction in the face-sensitive N170 is significantly associated with social dysfunction in patients with SZ (12, 13). To date, only one face recognition study in patients with SZ has used magnetoencephalography (MEG). Rivolta et al. (14) reported significantly increased magnetic M170 (a magnetic counterpart of the N170) responses during the perception of Mooney faces in antipsychotic-naïve first-episode patients with SZ; the authors concluded that this effect may indicate aberrant glutamatergic neurotransmission at illness onset (15). However, to our knowledge, no study has examined the specific face-sensitive M170 response in patients with chronic SZ. It has been suggested that reductions in glutamate levels observed in patients with SZ are caused by excitotoxicity during the acute phase of the illness, and that this may have a huge impact on patients’ brain function during recurrences throughout the course of the illness (15). Indeed, Tsai et al. (16) found decreased glutamate levels in the postmortem brains of patients with chronic SZ. Thus, given this evidence, we hypothesized that patients with chronic SZ would show reduced face-sensitive M170 responses that reflect altered glutamatergic neurotransmission in the chronic state.

MEG offers a higher spatial resolution than EEG, which allows the source of neuronal activity to be more accurately located. Furthermore, MEG waveforms cannot be deformed by conductivity affecting volume currents, which makes it more suitable for assessing the laterality of neural activity. In healthy subjects, it has been reported that the M170 is generated in the fusiform gyrus, which is a crucial region for facial recognition (17). However, Pierce et al. (18) reported that patients with autism process human faces outside the fusiform gyrus.

The present study examined, for the first time, the M170 response to faces in patients with chronic SZ, which can offer insights as to whether the fusiform gyrus is the center of face recognition in patients with chronic SZ. Given that patients with SZ have been reported to exhibit a bilateral N170 reduction that is specific to faces, we also investigated the M170 response to cars (see, for example, 13, 19). This work allows us to better understand whether the face recognition process is specifically impaired in chronic SZ. The present study was designed to test the hypothesis that patients with chronic SZ show less activation in early visual processing for faces than healthy subjects, as measured by the M170 response to faces.

## Materials and Methods

### Subjects

A total of 26 patients with SZ (13 male) and 26 healthy controls (HC; 16 male) aged between 20 and 65 years participated in the study. All participants were right-handed and had normal or corrected-to-normal visual acuity. After receiving a complete explanation of the study, all subjects signed an informed consent form according to the regulations of the Research Ethics Committee of the Graduate School of Medical Sciences, Kyushu University. For both groups, the exclusion criteria were as follows: 1) major head trauma; 2) neurological illness; 3) history of electroconvulsive therapy; 4) drug or alcohol dependence; 5) drug or alcohol abuse within the past five years; and/or 6) a verbal intelligence quotient below 75. The HC participants were screened using the Structured Clinical Interview (SCID) non-patient edition (20), which confirmed that no HC participants, or their first-degree relatives, had an Axis-I psychiatric disorder.

All patients were recruited from Kyushu University Hospital, the Japan Self-Defense Forces Fukuoka Hospital, and an affiliated private clinic (Kouno Clinic), and had been diagnosed with SZ based on medical records and the SCID-DSM IV (21). The patients’ symptoms were evaluated on the Scale for the Assessment of Positive Symptoms (SAPS) (22) and the Scale for the Assessment of Negative Symptoms (SANS) (23). **Table 1** shows the demographic data for all participants. All patients were receiving neuroleptic medication [typical neuroleptics (1/26 patients), atypical (20/26), or both (5/26)], with a mean daily dose equivalent to 398 ± 292 mg/day of chlorpromazine (24).

**Table 1:** Demographic and clinical characteristics of the subjects.

	HC	SZ	df	*t* or *x^2^*	*p*
Sex, M/F, No	16/10	13/13	1	0.70	0.40
Age (years)	40.4 ± 14.0	40.0 ± 10.8	50	0.12	0.90
Handedness	96.4 ± 10.3	99.4 ± 2.1	50	−1.32	0.20
SES	2.6 ± 0.9	3.3 ± 1.0	50	−2.53	0.02*
Parental SES	3.1 ± 0.8	2.8 ± 1.0	50	1.41	0.17
Medication dose (CPZ equiv., mg)		398 ± 292			
Symptom onset (years)		25.7 ± 9.0			
Duration of illness (years)		14.1 ± 9.9			
SAPS		33.2 ± 31.4			
SANS		54.4 ± 31.6			
GAF		56.5 ± 14.0			

The data are given as mean ± SD. HC, healthy controls; SZ, patients with schizophrenia. Asterisks indicate statistically significant results. SZ showed significantly lower SES than HC.

### Visual Stimuli and Experimental Procedures

The stimuli were two colored pictures of emotionally neutral faces (1 male and 1 female Asian faces), two pictures of cars, and one picture of a butterfly. Stimuli were presented using Stim2 software (Compumedics Neuroscan) video projector that was placed outside the magnetically shielded room in which the experiment was conducted. All images were 24 × 24 cm in size and the distance between the subjects’ eyes and the screen was 100 cm. During the experiment, the two faces and two cars were presented 60 times each, and the butterfly target was presented 30 times. Stimuli were presented in a pseudo-random order. The images appeared in the center of the screen for 500 ms, each preceded by a 200 ms interval during which a fixation point was presented in the middle of the screen at which subjects were asked to maintain their gaze, followed by a 200-ms interval during which the screen was blank. The interstimulus interval was 1,900 ms. Subjects were instructed to click a mouse button with their right hand as soon as they saw the target picture (butterfly). A no-response was recorded if the response time exceeded 1,500 ms.

### Data Collection and Processing

Magnetic signals were acquired using a 306-channel sensor array whole-head MEG system (Vectorview; ELEKTA Neuromag, Finland). The sampling rate was set to 1 kHz and the recording band-pass filter was set to 0.01–330 Hz. All data sets were filtered using Maxfilter to eliminate noise originating from outside of the brain (25). We analyzed data recorded using 204-sensor, planar-type gradiometers. Before the recording, four head position indicator (HPI) coils were attached to the scalp, and a three-dimensional digitizer was used to determine the anatomical landmarks of the head with regard to the HPI coils. The accurate location of the head with regard to the sensor array was measured using the HPI coils.

### Magnetic Global Field Power of the M170

**Figure 1:**
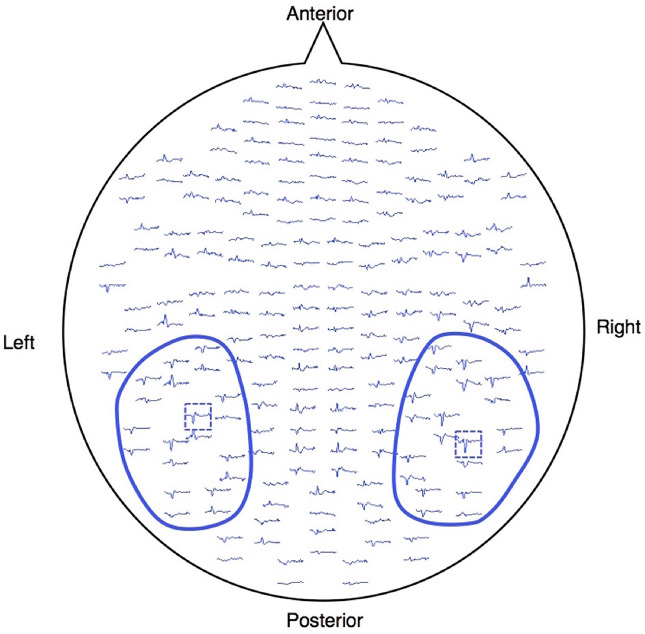
Waveforms of one healthy subject projected onto the measured 204 gradiometers. The MEG signals were acquired using a whole-head, 306-channel sensor array that comprised 102 identical triple-sensor elements. Each sensor consisted of two orthogonal planar-type gradiometers and one magnetometer. Circled waveforms with blue lines indicate the sensors used for the analysis. The blue dashed boxes are the sensors, which showed the largest response between 120 and 250 ms after the stimulus onset for each hemisphere.

The event-related field was calculated in each gradiometer by averaging artifact-free epochs (epochs larger than 2000 ft/cm were rejected) within a latency window of −100 to 700 ms, with 0 ms corresponding to the visual stimulus onset. The averaged waveforms were digitally filtered from 1 to 20 Hz using a band-pass Butterworth filter and then amplitude-baselined relative to the pre-stimulus period of −100 to 0 ms. To analyze the M170, we selected 10 pairs of sensors (20 sensors) around one gradiometer coil that showed the largest amplitude within 120–250 ms after stimuli onset in each temporal hemisphere (**Figure 1**), because signals are strongest when the sensors are located just above local cerebral sources (26). The magnetic counterpart of the global field power (mGFP) was obtained separately for each hemisphere using the root mean square (RMS) values across the selected 10 sensors. The peak amplitude of the M170 was defined as the largest amplitude of the individual mGFP within 120–250 ms after stimulus onset, and the M170 peak latency was defined as the time of the peak amplitude of M170 from stimulus onset.

### Source Localization Analysis

To investigate the distributed source of the M170, source localization analysis was performed for the averaged waveform before baseline correction using a minimum-norm estimation (27). We first conducted a co-registration between MEG data and a template MRI (MNI-305, 28) based on the head position data obtained through the HPI coils and 3-D digitizer (we excluded data from 6 HC and 8 SZ subjects from further source analysis because the co-registration accuracy was poor). We then computed the forward solutions for all source locations (mesh-patterned 8,196 dipole locations were marked in the standard brain) using a single-compartment boundary-element model. A noise covariance matrix was created using the pre-stimulus period epoch data. The noise-normalized source activity [dynamical statistical parametric mapping (dSPM); 29] at each source location was estimated from each time point of the averaged waveform using an MNE inverse operator computed from the forward solution and the noise covariance matrix. To visualize the distributed source of the M170 in each stimulus condition (faces or cars), we calculated the time-averaged dSPM values within 120–250 ms at each source location.

### Statistical Analysis

Student’s t-tests were used to assess between-group differences in age, handedness score, education years, SES, parental SES, and sleeping scale. The mGFP peak latencies and M170 amplitudes were analyzed using a repeated-measures analysis of variance (rmANOVA), with group (SZ or HC) as a between-subjects factor, and hemisphere (left or right) and stimulus type (faces or cars) as within-subjects factors. Degrees of freedom were adjusted using the Huynh-Feldt epsilon for factors with more than two levels. The associations between the clinical symptoms and the mGFP peak latencies and powers of the M170 and dipole moments were investigated using Spearman’s rho. All results were considered significant at p ≤ 0.05.

## Results

### Demographics

There were no significant between-group differences in age, handedness, education years, or sleeping scale. The SZ group had a significantly lower SES and parental SES than the HC group. The SZ group had a significantly longer mean reaction time than the HC group (t[50] = −2.94, p = 0.05), but there were no between-group group differences in the accuracy of target responses (SZ, 97.7%; HC, 97.1%; t[50] = −0.39, p = 0.70).

### Magnetic Global Field Power of the M170

**Figure 2:**
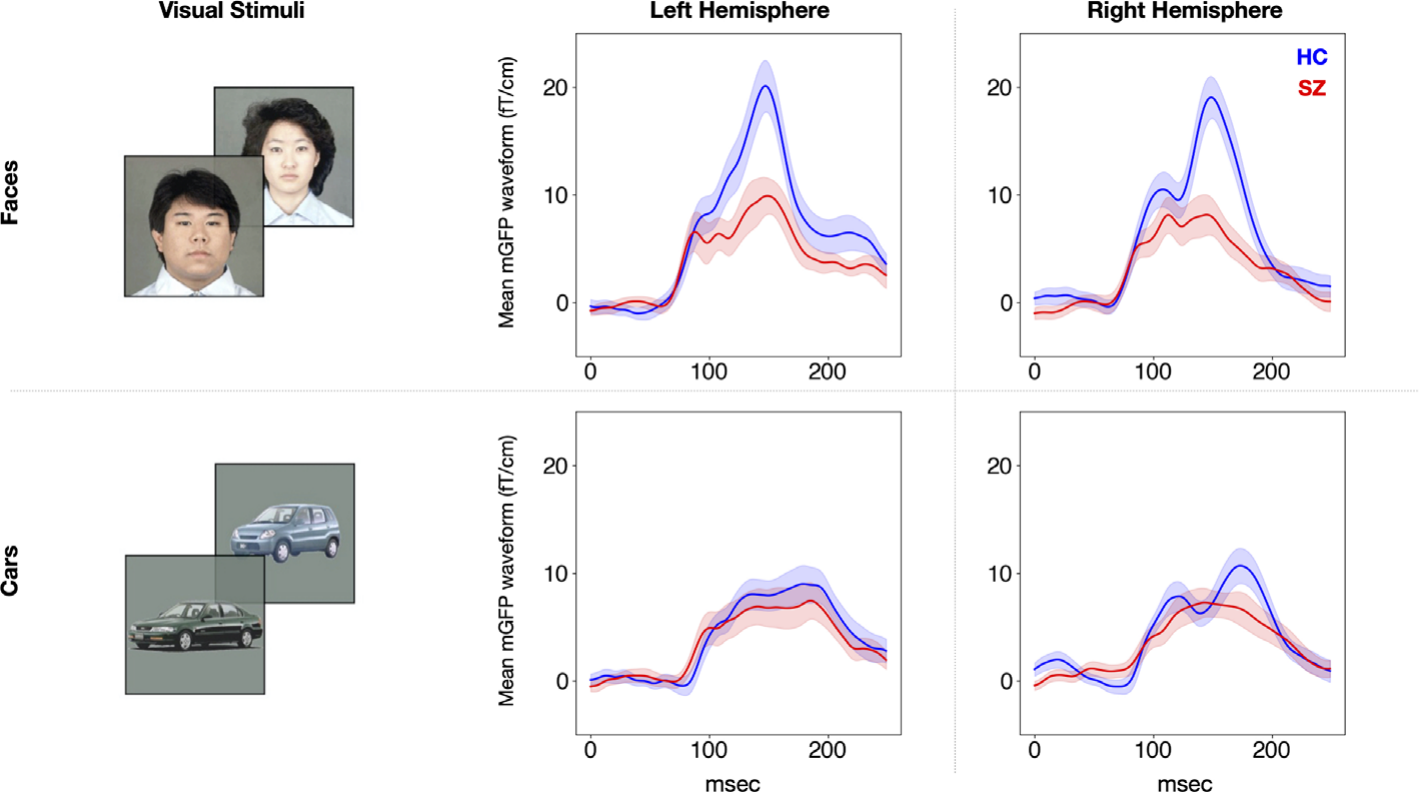
Group average M170 waveforms in response to faces and cars for each hemisphere. The waveforms of the SZ group (n = 26) are shown in red and those of the HC group (n = 26) are shown in blue. The SEM is plotted semitransparent. SZ, patients with schizophrenia; HC, healthy control.

The rmANOVA revealed a significant main effect of group [F (1,50) = 7.1, p = 0.01] and stimulus [F (1,50) = 31.3, p < 0.001] on the mGFP value of the M170 (**Figure 2**). There was also a significant stimulus-by-group interaction effect [F (1,50) = 14.6, p < 0.001]. There was no main effect of hemisphere on the mGFP value of the M170 [F (1,50) = 0.9, p = 0.36] and no interactions related to group (0.56 ≤ p ≤ 1.00). To delineate the significant stimulus-by-group interaction, stimulus differences and group differences were compared using t-tests using the average power of M170 of both hemispheres. In the HC group, the M170 power in response to face stimuli was significantly greater than that in response to cars (t[25] = 5.3, p < 0.001), but the SZ group showed no such significant differences (t[25] = 0.9, p = 0.38). The SZ group showed a significant M170 power reduction in response to faces compared to the HC group (t[50] = 3.7, p = 0.01). However, there were no significant between-group differences in response to cars (t[50] = 0.6, p = 0.55). These results suggest that the SZ group showed an M170 reduction that was specific to faces.

### Latency of the M170

**Table 2** shows the latencies of the M170. The rmANOVA revealed a significant main effect of stimulus on M170 latency [F (1,50) = 35.6, p < 0.001]. There was no main effect of group and no group-related interactions.

**Table 2:** Mean M170 power and latencies averaged in healthy controls (N = 26) and patients with schizophrenia (N = 26).

	Face					Car				
	HC	SZ	df	t	p	HC	SZ	df	t	p
**Amplitude (fT/cm)**										
Right	23.2 ± 10.4	13.3 ± 9.6	50	3.5	0.001	13.4 ± 7.3	12.6 ± 8.2	50	0.33	0.74
Left	24.6 ± 12.6	15.6 ± 10.5	50	2.8	0.008	14.2 ± 8.0	12.6 ± 9.5	50	0.65	0.52
**Latency (ms)**										
Right	149.7 ± 11.9	156.5 ± 20.7	50	−1.4	0.16	168.9 ± 19.0	169.4 ± 21.4	50	−0.10	0.92
Left	155.7 ± 21.5	161.9 ± 22.2	50	−1.0	0.31	170.8 ± 24.8	172.1 ± 24.0	50	−0.19	0.85

The data are shown as mean ± SD.

HC, healthy controls; SZ, individuals with schizophrenia.

### Source Distribution of the M170

**Figure 3:**
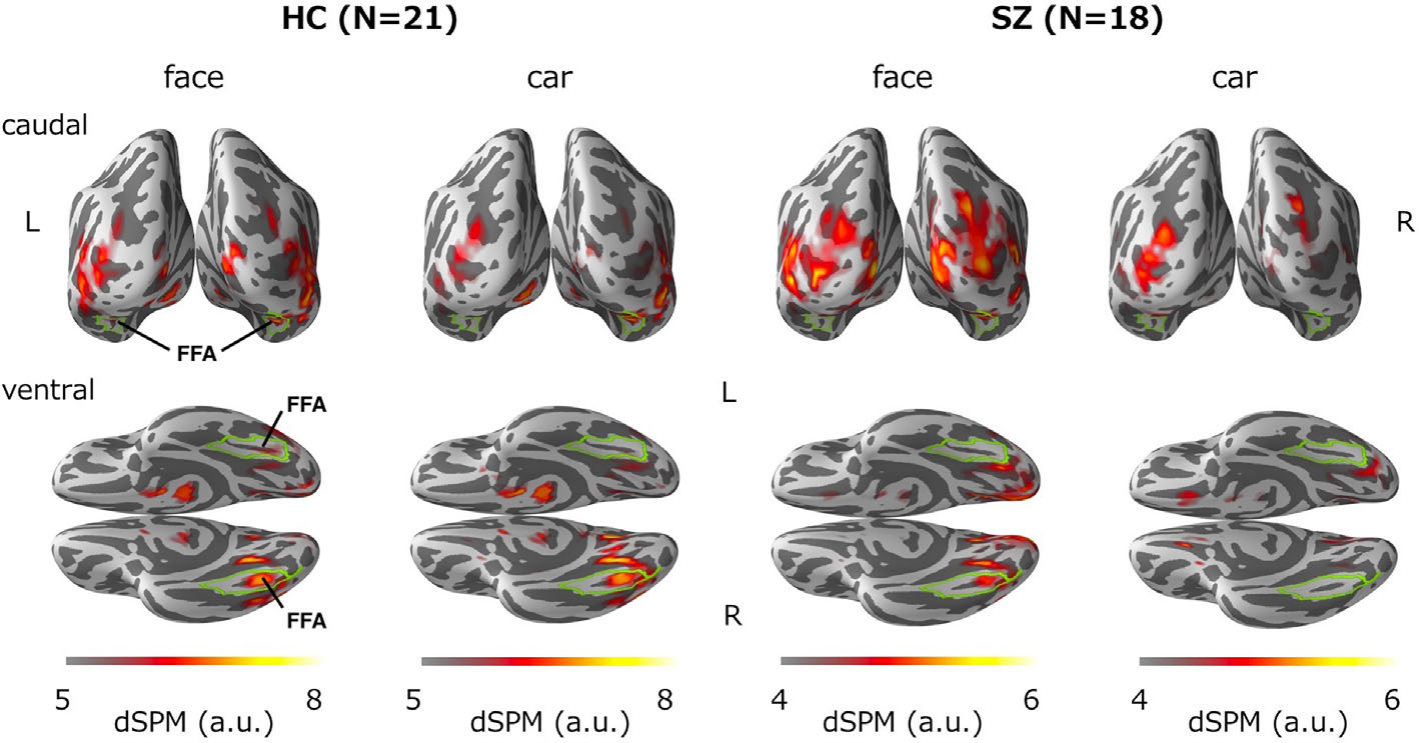
Group-averaged source distribution maps of the M170 in response to faces and cars. The upper and lower panels show the distribution maps from the caudal and ventral views, respectively. The horizontal bars at the bottom indicate the colored threshold levels of the distribution maps. The fusiform gyrus is outlined in green. FFA, fusiform face area.

**Figure 3** shows group-averaged source distribution maps of the M170 in responses to faces and cars. In the HC group, strong activations were observed near or within the fusiform face area (FFA) of the right hemisphere in both stimulus conditions. In contrast, source activity spread broadly throughout visual-related brain regions other than the FFA in the SZ group.

### Correlations Between M170 Power and Clinical Measurements

**Figure 4:**
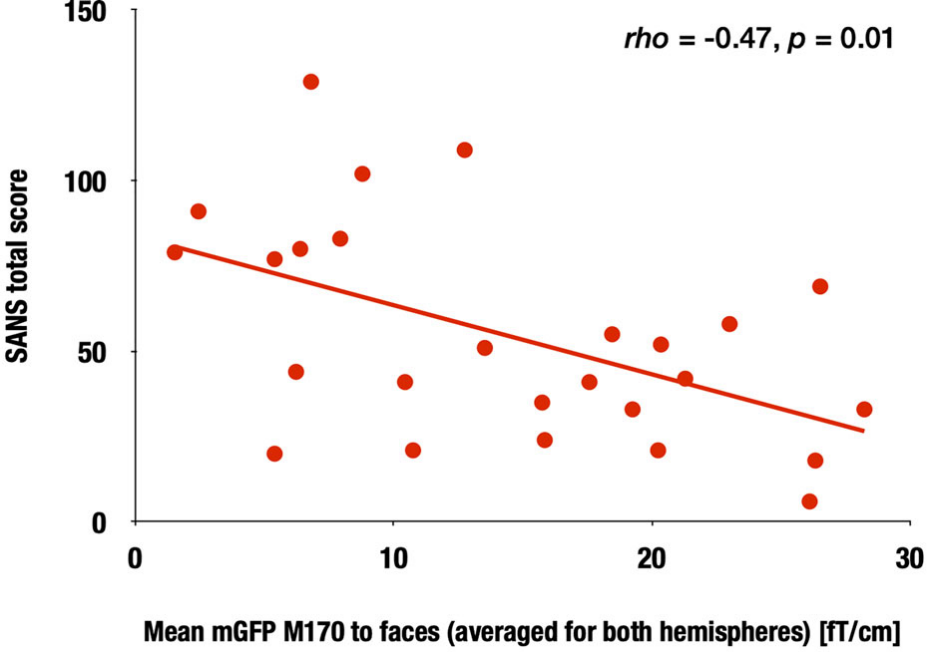
Correlation between the M170 response to faces and SANS total scores in the SZ group. Scattergram showing the relationship between the M170 response to faces averaged for both hemispheres and the SANS total scores in the SZ group.

In the SZ group, we calculated the correlation between the face-evoked M170 power averaged across both hemispheres, as well as within the left and right hemispheres, and the clinical symptom scores. The M170 power was significantly correlated with the SANS total score (averaged: rho = −0.47, p = 0.01; left: rho = −0.46, p = 0.02; right: rho = −0.42, p = 0.04; **Figure 4**). There were no significant correlations between demographic or symptom scores and M170 variables in response to any stimuli (−0.37 ≤ rho ≤ 0.30, 0.06 ≤ p ≤ 0.93). There were no significant correlations between chlorpromazine-equivalent doses and the value of the M170 in response to any stimuli (−0.19 ≤ rho ≤ 0.28, 0.19 ≤ p ≤ 0.94).

**Figure 5:**
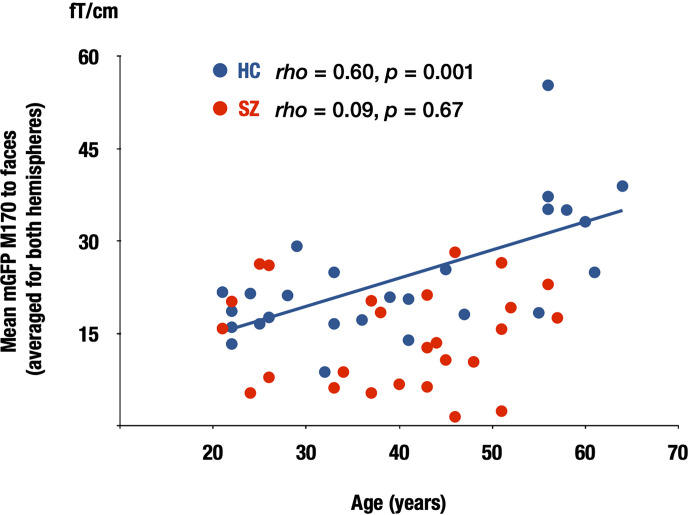
Correlation between the M170 response to faces and age in both groups. Scattergrams showing the relationship between the M170 response to faces averaged for both hemispheres and age in the HC and SZ groups. SZ: patients with schizophrenia (red), HC, healthy control (blue).

Within the HC group, there was a significant correlation between age and the M170 global field power to faces averaged for both hemispheres (rho = 0.60, p = 0.001), while such a relationship was not observed in the chronic SZ group (rho = 0.09, p = 0.67; **Figure 5**).

## Discussion

The major findings of this study were as follows: 1) the power of the M170 was significantly smaller in SZ group than in the HC group, specifically in response to human faces, 2) there was no significant between-group difference in the dipole locations of the M170, which were estimated to originate from the fusiform gyrus, 3) SANS scores were negatively correlated with M170 power to faces, and 4) for the HC group, there was a significant correlation between age and the M170 power to faces, while such a relationship was not observed in the chronic SZ group.

Our finding of an M170 reduction in response to faces in patients with chronic SZ is consistent with the face-sensitive N170 attenuations reported in a meta-analysis (10) and a recent systematic review (30). The meta-analysis reported that the effect size of the face-sensitive N170 reduction was 0.64 (10). For first-episode or drug-naïve patients with SZ, the small number of studies that have investigated the N170 or M170 response to faces have reported mixed findings. For example, Rivolta et al. (14) reported a significantly increased magnetic face-sensitive M170 to pictures of moony faces in antipsychotic-naïve, first-episode patients with SZ. Yang et al. (31) reported no N170 reduction in response to emotional faces in first-episode/early course (<3 years of illness) patients with SZ. However, Salisbury et al. (32) showed a significant N170 attenuation in response to neutral faces and cars in first-hospitalized patients with SZ. Tsunoda et al. (13) found a significant N170 reduction in response to neutral faces in the early course of SZ (the average duration of illness: 6.4 months). The samples of Salisbury et al. (32) and Tsunoda et al. (13) were medication managed. Therefore, the effects of acute psychosis and medication on face processing should be further examined.

Although it is not yet clear whether the face-sensitive M170 is altered in acute drug-naïve patients, the previous and the current findings indicate that the face-sensitive M170 is decreased in patients with chronic medicated SZ. Rivolta et al. (14) have suggested that a shift of the excitation/inhibition balance toward excitation could lead to face-sensitive M170 abnormalities. Reduced face-sensitive M170 responses found in the current study might be associated with altered glutamatergic neurotransmission in the chronic state, with possible progressive pathophysiological changes after the onset of SZ.

It is not yet clear why face-sensitive N170 and M170 responses are specifically impaired in chronic SZ compared to the processing of other visual objects. In social communication, people need to distinguish human faces instantaneously. It may be possible that sophisticated processes such as face recognition may be more vulnerable to abnormal neuronal excitation, while general visual processing deficits progress slowly after the onset of SZ. Salisbury et al. (32) reported significant N170 reductions in response to faces and cars in SZ. They argued that their sample size was large in comparison with previous studies and suggested that possible power issues partially allowed them to detect overall N170 reduction in SZ. A comprehensive review has pointed out that, at the behavioral level, visual perception deﬁcits in SZ may not be speciﬁc to faces, are often present when the cognitive and perceptual demands of a task are high, and are likely to worsen with the progression of the illness (33). Thus, further research is required to clarify our findings at both the behavioral and neurophysiological levels.

The present study found that a face-sensitive M170 reduction is associated with dysfunction of the fusiform gyrus in chronic SZ. Onitsuka et al. (19) found a significant association between face-sensitive N170 attenuation and gray matter reduction of the fusiform gyrus in the right hemisphere of patients with chronic SZ. Thus, both structural and functional deficits could underlie this face-sensitive attenuation in SZ. At the cellular level, the face-sensitive M170 deficit identified in our study may involve progressive dendritic spine alterations (34), which is the strongest source of evoked responses (35). Furthermore, in terms of the source distribution of M170, the MNE analysis demonstrated that while the M170 was localized over the FFA in the HC group, visual-related brain regions other than the FFA were strongly activated in the SZ group in both stimulus conditions (**Figure 3**). Thus, it is likely that face-sensitive M170 abnormality could arise from inefficient neural network activities during visual face processing in SZ.

We found a shorter M170 latency for faces than for cars in both groups, which was compatible with our previous findings (e.g., 13). The N170/M170 are known to show larger and earlier peak activity in response to face stimuli than to non-face stimuli (36–38). Hence, our results suggest that while face-sensitive M170 power is decreased, its processing speed remains normal in SZ.

We found that patients with chronic SZ with more severe negative symptoms had smaller face-sensitive M170 responses, which is generally consistent with the literature. However, it should be noted that the sample size for our correlational analyses may not be sufficient, which needs to be replicated in more extensive sample studies. One recent systematic review (30) reported a tendency for an association between a face-sensitive N170 reduction and higher severity of positive and negative symptoms. The authors suggested that the face-sensitive N170 may be useful for evaluating social functioning and rehabilitation efficacy. In patients with temporal lobe epilepsy, one study found that socioeconomic status was significantly correlated with the N170 amplitudes to faces (39). The present study did not demonstrate specificity to schizophrenic psychosis, as we did not include another psychosis group. Therefore, the M170 in other psychoses or neuropsychiatric diseases should be further examined. Moreover, given that longitudinal studies have highlighted the use of EEG/MEG indices as biomarkers for detecting the onset and risk of SZ (40–43), it could be important to evaluate the longitudinal face-specific M170/N170 changes during the early phase of SZ in future studies. Further, long-term test-retest reliability studies (44) may confirm the robustness of the M170/N170 index as a biomarker. Since face and language processing deficits (45) in SZ may interact with each other, it is vital to investigate the neural integration mechanism during simultaneous voice-face information processing (46) in patients with SZ in future. Investigating the association between neurophysiological deficits and structural deficits (e.g., 19, 47) may also shed light on the underlying mechanism of this face processing abnormality.

In the present study, there was a significant association between age and the M170 power to faces in the HC group. It has been reported that the N170 amplitudes in response to faces were larger in older than in young healthy participants (48–52), and the current result is consistent with previous findings. For patients with chronic SZ, such a relationship was not observed. These findings suggest that the normal aging process may not occur in patients with SZ.

## Conclusion

In summary, the present study showed specific M170 reductions in response to human faces in patients with chronic SZ, and SZ may be characterized by face processing deficits associated with the severity of negative symptoms. We suggest that social cognition impairments in SZ might, at least in part, be caused by functional face processing deficits. Thus, face-specific M170 activity could be a useful biological index to evaluate social cognition abnormalities in SZ.

## Data Availability Statement

The raw data supporting the conclusions of this article will be made available by the authors, without undue reservation.

## Ethics Statement

The studies involving human participants were reviewed and approved by The Ethics Committee of the Graduate School of Medical Sciences, Kyushu University. The patients/participants provided their written informed consent to participate in this study. Written informed consent was also obtained from the individuals for the publication of any potentially identifiable images or data included in this article.

## Author Contributions

TO and NOh designed the study. NOh, NOr, and IN collected and analyzed the data. NO prepared the first draft of the manuscript. YH, NOr, SH, RT, and ST assisted data analyses and created figures. TU, OT, AH, TN, and ST edited the manuscript. YH and TO critically revised manuscript. All authors contributed to and have approved the final manuscript.

## Funding

This research was supported, in part, by AMED under Grant Number JP20dm0207069 (TO); Grant-in-Aid for Young Scientists B JP22791129 (YH), JP 15K19735 (NOr), and JP 17K16385 (NOr), a Grant-in-Aid for Scientific Research C: JP 16K10217 (TO), JP 19K08049 (NOr), JP 15K09836 (YH), JP 18K07604 (YH), JP 19H03579 (YH) from the Japanese Ministry of Education, Culture, Sports, Science, and Technology; Medical Research Fund (YH) from Takeda Science Foundation; SIRS Research Fund Award (YH) from Schizophrenia International Research Society. The funding sources had no further role in study design; in the collection, analysis, and interpretation of data; in the writing report; and in the decision to submit the paper for publication.

## Conflict of Interest

The authors declare that the research was conducted in the absence of any commercial or financial relationships that could be construed as a potential conflict of interest.

The reviewer, TM, declared a shared affiliation, though no collaboration, with the authors to the handling editor.
